# Optimizing the osteo-immunomodulatory balance: threshold-saturation effects of BMP-2-loaded allografts in the Masquelet’s induced membrane technique (MIMT)

**DOI:** 10.3389/fbioe.2026.1818741

**Published:** 2026-06-11

**Authors:** Junyi Li, Kehan Lv, Yunan Li, Hangyu Li, Guohao Liu, Wei Zhang, Xingyu Chen, Muguo Song, Jian Shi

**Affiliations:** 1 Department of Orthopaedics, 920 Hospital of the Joint Logistics Support Force of the PLA, Kunming, China; 2 Yunnan Province Key Laboratory of Microsurgical Medical Technology, Kunming, China

**Keywords:** allograft, bone defect, bone morphogenetic protein-2 (BMP-2), dose-response, masquelet, systemic inflammation

## Abstract

**Background:**

The Masquelet induced membrane technique (MIMT) is an established strategy for reconstructing critical-sized bone defects (CSDs). However, the limited osteoinductivity of allografts often necessitates supplementation with bone morphogenetic protein-2 (BMP-2), whose supra-physiological doses may cause excessive inflammation. This study aimed to identify a BMP-2 concentration range that balances osteogenic efficacy with systemic safety in the MIMT setting.

**Methods:**

A 1.5-cm critical-sized radial defect was created in 36 New Zealand white rabbits. In Stage I, a polymethyl methacrylate spacer was implanted to induce membrane formation. In Stage II, performed 4 weeks later, the spacer was removed and the defect was filled with freeze-dried allograft standardized to the nominal defect volume, calculated by the cylindrical formula (V = πr^2^L). Using a radius of 0.2 cm and a length of 1.5 cm, the nominal defect volume was 0.188 cm^3^ (≈0.188 mL). BMP-L, BMP-M, and BMP-H allografts were loaded with rhBMP-2 at 5, 10, and 20 μg mL^-1^, corresponding to nominal doses of 0.94, 1.89, and 3.77 µg per defect; the Allograft group received unloaded allograft. Bone repair was assessed by serial X-ray at 2, 4, 8, 12, and 16 weeks, by micro-CT at 2, 8, and 16 weeks, and by histological and immunohistochemical analyses up to 16 weeks. Systemic inflammatory and reparative dynamics were evaluated at 12 timepoints by ELISA.

**Results:**

At 16 weeks, radiographic and micro-CT analyses showed that BMP-M and BMP-H achieved significantly higher bone mineral density and BV/TV than BMP-L and allograft alone (P < 0.05). Osteogenic marker expression increased dose-dependently from 0 to 10 μg mL^-1^. However, increasing the dose from 10 to 20 μg mL^-1^ did not yield meaningful additional benefit in cortical bridging or trabecular maturation. The BMP-H group also showed an early post-Stage II inflammatory rebound in serum CRP and IL-6, whereas BMP-M maintained a more stable systemic immune profile with a higher IL-10/IL-6 ratio.

**Conclusion:**

Within the tested conditions, 10 μg mL^-1^ BMP-2 appeared to provide the most favorable balance between bone regeneration and systemic inflammatory stability in MIMT.

## 1 Introduction

Critical-sized bone defects (CSDs) remain a formidable challenge in reconstructive orthopedics, particularly when arising from high-energy trauma, tumor resection, or chronic osteomyelitis (Helbig et al., 2020; Hettwer et al., 2019). These defects exceed the innate regenerative capacity of bone, leading to prolonged healing cycles, high recurrence rates, and significant socioeconomic burdens (Helbig et al., 2020; Yang et al., 2024).

Among various reconstructive strategies, Masquelet’s induced membrane technique (MIMT) has emerged as a widely accepted reconstructive strategy for long-bone segmental reconstruction (Masquelet, 2020). This two-stage procedure utilizes a polymethyl methacrylate (PMMA) spacer to trigger a localized foreign-body response, forming a biological membrane rich in osteogenic progenitors and growth factors (e.g., BMP-2, TGF-β1, VEGF) (Masquelet and Begue, 2010; Wang et al., 2015). While the autologous cancellous bone graft remains the preferred filling material for the second stage, its clinical utility is often hampered by limited donor-site availability and potential morbidity, especially in CSD exceeding 5–6 cm (Masquelet et al., 2019). Allografts offer a viable alternative for increasing graft volume; however, conventional freeze-dried allografts primarily provide osteoconductive support and generally exhibit limited intrinsic osteoinductive activity compared with autograft. Current consensus suggests that bone healing may be compromised when the allograft proportion exceeds one-third of the total graft volume (Mathieu et al., 2022). Consequently, augmenting allografts with exogenous growth factors is essential to overcome the limitations of MIMT in treating CSDs.

Bone morphogenetic protein-2 (BMP-2) is a potent orchestrator of bone regeneration, promoting mesenchymal stem cell differentiation via the Runx2 signaling pathway and synergizing with VEGF to enhance angiogenesis (Yu et al., 2018; Rady et al., 2020). Despite its efficacy, the delivery of BMP-2 in CSDs faces a “dose-effect paradox.” Due to rapid systemic clearance and limited carrier retention, supra-physiological doses (often in the milligram range) are frequently employed in clinical practice-doses that exceed endogenous levels by a million-fold (Lo et al., 2012; Sanchez-Casanova et al., 2020). Such excessive concentrations are associated with severe complications, including exuberant inflammatory responses, ectopic ossification, hematomas, and potential tumorigenicity (Sanchez-Casanova et al., 2020; Nguyen et al., 2017; Wang et al., 2012). Within the specific microenvironment of the Masquelet induced membrane, the optimal exogenous BMP-2 concentration that balances osteogenic potency with immunological safety remains poorly defined.

In this study, we established a 1.5 cm critical-sized radial defect model in rabbits to systematically evaluate the dose-dependent effects of BMP-2-loaded allografts within the MIMT framework. By integrating longitudinal radiologic assessment, histological maturation grading, and systemic inflammatory cytokine profiling (CRP, IL-6, TNF-α, and IL-10), we aimed to explore a potentially favorable dosing range for BMP-2 application. Our findings seek to provide an experimental foundation for refining BMP-2 dosing strategies to maximize bone healing while minimizing adverse inflammatory risks in complex bone reconstructions.

## 2 Materials and methods

### 2.1 Materials and reagents

The freeze-dried allograft bone was provided by Biogin Co., Ltd. (China). Polymethyl methacrylate (PMMA) bone cement was provided from Heraeus (Germany). Recombinant human BMP-2 (rhBMP-2) was obtained from MedChem Express (MCE, China). Primary antibodies, including rabbit anti-collagen I, anti-RUNX2, anti-osteocalcin (OCN), and anti-osteopontin (OPN), were also sourced from MedChem Express (NJ, United States). Masson’s trichrome staining kits and other histological reagents were purchased from Beyotime (China).

### 2.2 Animal grouping and ethical approval

Thirty-six male New Zealand white rabbits (3–6 months old; body weight, 3.0 ± 0.2 kg) were obtained from the Laboratory Animal Center of Kunming Medical University. All animal procedures were approved by the Ethics Committee of the 920th Hospital of the Joint Logistics Support Force of the PLA (Approval No. 2025–067(K)-01) and were conducted in accordance with the Guide for the Care and Use of Laboratory Animals. This study is reported in accordance with the ARRIVE 2.0 guidelines.

After a 3-day acclimatization period, animals were allocated to six groups using computer-generated randomization: Blank Control group (defect without treatment), Sham group (surgical exposure without ostectomy), Allograft group (allograft alone), BMP-L group (allograft + rhBMP-2 at 5 μg mL^-1^), BMP-M group (allograft + rhBMP-2 at 10 μg mL^-1^), and BMP-H group (allograft + rhBMP-2 at 20 μg mL^-1^). Sample size was determined based on pilot data and G*Power-based estimation, with a minimum of five animals per group and one additional animal per group to account for possible attrition. Outcome assessments, including radiographic scoring, micro-CT quantification, histological evaluation, and immunohistochemical quantification, were performed by investigators blinded to group allocation. Animals were monitored daily for wound condition, activity, feeding behavior, and signs of distress or infection. Humane endpoints included persistent weight loss >20% from baseline, marked reduction in spontaneous activity, self-injurious behavior or refusal to feed for >24 h, severe respiratory distress, or uncontrollable bleeding.

### 2.3 The two-stage MIMT

**Stage I:** Rabbits were anesthetized with an intravenous injection of 3% pentobarbital (30 mg/kg). Under sterile conditions, a 1.5 cm critical-sized segmental defect was created in the mid-shaft of the radius. All bone defects except the blank control group were filled with a polymethyl methacrylate (PMMA) bone cement spacer for induced membrane formation.

**Stage II:** Four weeks later, the induced membrane was carefully incised and the PMMA spacer was removed. The graft volume was standardized according to the geometric volume of the original cylindrical defect $\left(V=\pi r^{2}L\right)$ . Using a defect radius of 0.2 cm and a defect length of 1.5 cm, the nominal defect volume was calculated as approximately 0.188 cm^3^ (≈0.188 mL). Freeze-dried allograft was prepared to match this nominal defect volume and implanted into the membrane chamber. For the BMP-L, BMP-M, and BMP-H groups, the allograft was loaded with rhBMP-2 solutions at concentrations of 5, 10, and 20 μg mL^-1^, respectively, by soaking for 2 h followed by vacuum drying before implantation. Based on the standardized defect volume, the corresponding nominal rhBMP-2 amount per defect was 0.94, 1.89, and 3.77 µg, respectively. Internal fixation was performed using a 1.2-mm Kirschner wire.

### 2.4 Radiographic evaluation and lane-Sandhu scoring

Digital X-ray images were obtained at 2, 4, 8, 12, and 16 weeks after Stage II surgery. The progression of bone union, callus formation, and cortical bridging was independently evaluated by two radiologists blinded to group allocation using the Lane-Sandhu scoring system, which assesses bone formation, proximal union, and distal union.

### 2.5 Micro-computed tomography (Micro-CT) analysis

At 2, 8, and 16 weeks after Stage II surgery, the harvested radii were fixed and scanned using a high-resolution micro-CT system (SkyScan 1276, Bruker, Germany). Scanning was performed at 85 kV and 200 μA with an isotropic voxel size of 9.065 μm, an exposure time of 240 m, a rotation step of 0.25°, and a 180° rotation, using a 1-mm Al filter. A manufacturer-provided phantom was scanned under identical conditions for calibration. Raw projection images were reconstructed using NRecon software (version 1.7.4.2, Bruker), and quantitative analysis was performed using CTAn software (version 1.20.3.0, Bruker). The region of interest (ROI) encompassed the original 1.5-cm defect region between the two host-bone ends and excluded the ulna and fixation-related artifacts. A uniform grayscale threshold greater than 1000 was applied for segmentation of mineralized tissue in all specimens. Quantitative parameters, including total volume (TV), bone volume (BV), bone volume fraction (BV/TV), trabecular number (Tb.N), and trabecular thickness (Tb.Th), were calculated to evaluate the structural characteristics of the regenerated tissue. Quantitative micro-CT analysis was performed by an investigator blinded to group allocation.

### 2.6 Histological and immunohistochemical (IHC) analysis

The harvested specimens were fixed in 10% neutral buffered formalin, decalcified in 10% EDTA (pH 7.4) at 4 °C, embedded in paraffin, and sectioned at 3–6 μm. H&E and Masson staining were performed to evaluate tissue morphology, collagen deposition, and graft incorporation. For immunohistochemistry, RUNX2, Collagen I, OPN, and OCN were assessed using standard procedures, and images were analyzed with Image-Pro Plus 6.0. Three representative fields from each sample were selected for semiquantitative analysis. Semiquantitative analysis was performed according to positive staining area within the selected regions of interest for RUNX2, Collagen I, OPN, and OCN. Histological evaluation and immunohistochemical quantification were performed by investigators blinded to group allocation.

### 2.7 Serum cytokine dynamics (ELISA)

1.5 mL of auricular vein blood was collected at 12 timepoints: T_0_ (baseline); T_1_–T_5_ (6 h, 1 d, 3 d, 7 d, 14 d post-Stage I); T_6_–T_9_ (1 d, 3 d, 7 d, 14 d post-Stage II); and T_10_–T_11_ (8 w, 16 w post-Stage II). Serum levels of pro-inflammatory (CRP, IL-6, TNF-α, IL-1β) and anti-inflammatory/reparative (IL-10, TGF-β_1_) factors were measured using double-antibody sandwich ELISA kits (Elabscience, China) according to the manufacturer’s instructions. The IL-10/IL-6 ratio was calculated to reflect the systemic immune homeostasis.

### 2.8 Statistical analysis

Data are expressed as mean ± standard deviation (SD). Comparisons between multiple groups were performed using one-way analysis of variance (ANOVA) followed by Tukey’s *post hoc* test for multiple comparisons. Statistical analysis was conducted using GraphPad Prism 8.0 (GraphPad Software, United States). A *P*-value <0.05 was considered statistically significant.

## 3 Results

### 3.1 General observations and radiographic assessment

**FIGURE 1:**
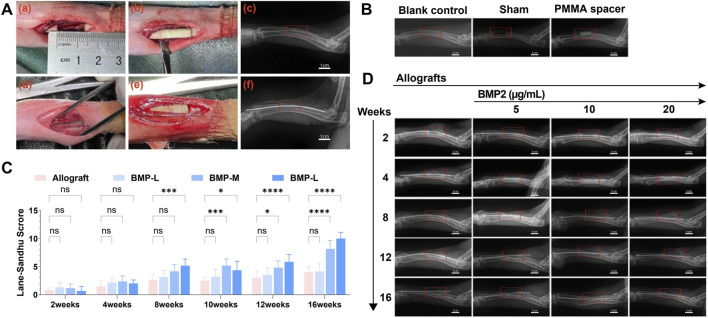
General observation and radiographic evaluation of radial bone defect repair. **(A)** Experimental procedure: (a) creation of a 1.5-cm radial defect; (b) insertion of a PMMA spacer during Stage I; (c) postoperative radiograph after Stage I; (d) re-exposure of the defect during Stage II; (e) implantation of allograft with or without rhBMP-2; and (f) postoperative radiograph after Stage II. **(B)** Representative radiographs at 16 weeks in the blank control, sham, and PMMA spacer groups. **(C)** Lane–Sandhu radiographic scores at 2, 4, 8, 12, and 16 weeks. **(D)** Serial radiographs of the allograft, BMP-L, BMP-M, and BMP-H groups at 2, 4, 8, 12, and 16 weeks after Stage II surgery. Red boxes indicate the original defect region. Scale bars = 1 cm. BMP-L, BMP-M, and BMP-H correspond to 5, 10, and 20 μg mL^-1^ rhBMP-2, respectively. Data are presented as mean ± SEM (n = 6). ns, not significant; *P < 0.05; ***P < 0.001; ****P < 0.0001.

All experimental animals survived the surgery without signs of infection or fixation failure. Postoperative X-rays confirmed precise graft placement (Figure 1A). Serial radiographic follow-up was performed at 2, 4, 8, 12, and 16 weeks after Stage II surgery. The Blank Control (BC) and Sham groups showed no spontaneous bone union, with persistent radiolucent gaps and the PMMA spacer maintaining a high-density signal at 16 weeks (Figure 1B). In contrast, rhBMP-2 groups (BMP-L, BMP-M and BMP-H groups) exhibited a dose-dependent acceleration of bone healing (Figure 1D). Sparse callus appeared at 2 weeks in all BMP-2 groups. By 8 weeks, the BMP-M group formed continuous cortical bridges, while the BMP-H group showed near-complete defect filling. At 16 weeks, both the BMP-M and BMP-H groups achieved solid union and medullary reconstruction, whereas the BMP-L group still exhibited partial gaps. Quantitative Lane-Sandhu scores confirmed that 10–20 μg mL^-1^ rhBMP-2 significantly enhanced radiographic repair quality compared to lower doses (Figure 1C).

### 3.2 Micro-CT evaluation of bone volume and density

**FIGURE 2:**
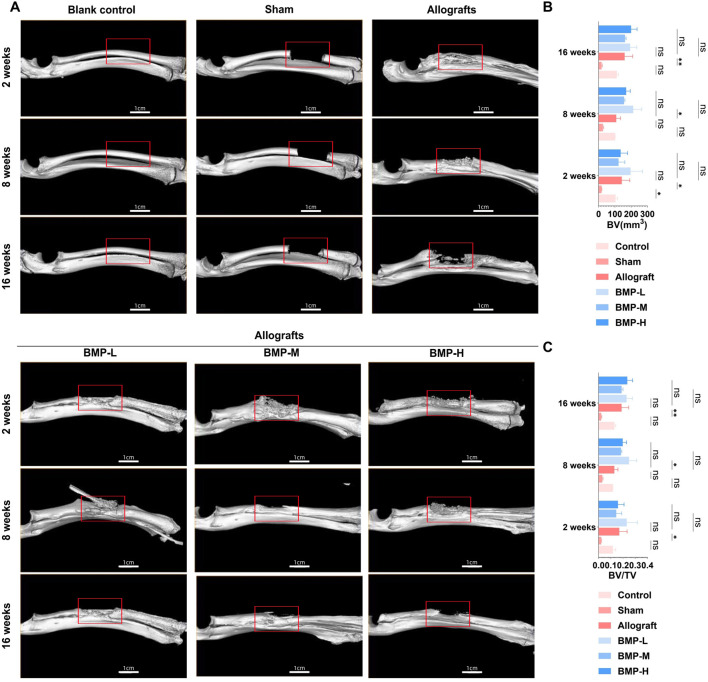
Micro-CT evaluation of radial defect repair. **(A)** Representative three-dimensional micro-CT reconstructions of the blank control, sham, and allograft groups at 2, 8, and 16 weeks. Red boxes indicate the original defect region. Scale bars = 1 cm. **(B)** Quantitative analysis of bone volume (BV) within the defect region at 2, 8, and 16 weeks. **(C)** Quantitative analysis of bone volume fraction (BV/TV) within the defect region at 2, 8, and 16 weeks. **(D)** Representative three-dimensional micro- CT reconstructions of the BMP-L, BMP‐M, and BMP-H groups at 2, 8, and 16 weeks. Red boxes indicate the original defect region. Scale bars = 1 cm. Data are presented as mean ± SEM. ns, not significant; *P < 0.05; **P < 0.01. BMP-L, BMP-M, and BMP-H correspond to 5, 10, and 20 μg mL-1 rhBMP-2, respectively.

3D reconstructions revealed minimal bone formation in the BC and Sham groups throughout the 16-week period (Figure 2A). In rhBMP-2 groups, bone volume (BV) and bone volume fraction (BV/TV) increased significantly from 8 to 16 weeks (Figures 2B,C). By 16 weeks, both BMP-M and BMP-H groups reached a BV/TV plateau (approximately 0.30), showing homogenous bone plates and advanced medullary reconstruction. Notably, while the BMP-M group was significantly superior to the BMP-L group (*P* < 0.05), increasing the dose to BMP-H yielded no significant additional gain in BV/TV. This “threshold-saturation” effect suggests that 10 μg mL^-1^ is sufficient to maximize bone quantity within the MIMT framework.

### 3.3 Histological assessment of bone maturity and remodeling

**FIGURE 3:**
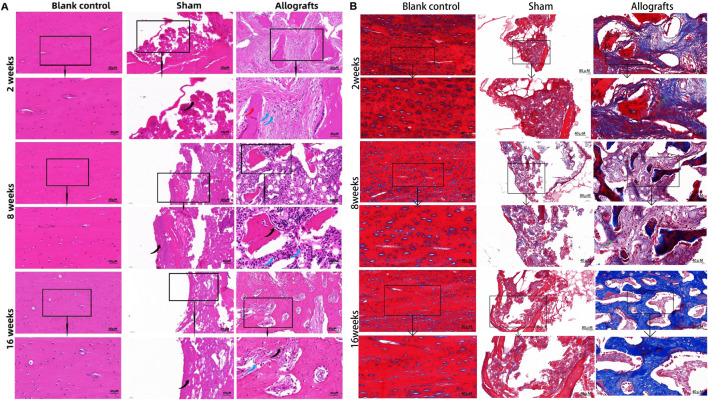
Histological evaluation of radial defect repair. **(A)** Representative H&E-stained sections of the blank control, sham, allograft, BMP-L, BMP-M, and BMP-H groups at 2, 8, and 16 weeks. For each specimen, a low-magnification overview is shown above and the corresponding high-magnification image of the boxed region is shown below. **(B)** Representative Masson’s trichrome-stained sections of the same groups and timepoints, displayed in the same format. Black boxes indicate the regions selected for higher-magnification imaging. Black arrows indicate osteoblasts, blue arrows indicate inflammatory cell infiltration, and green arrows indicate collagen fibers. Scale bars = 80 μm in overview images and 40 μm in higher-magnification images. BMP-L, BMP-M, and BMP-H correspond to 5, 10, and 20 μg mL^-1^ rhBMP-2, respectively.

H&E staining (Figure 3A) showed that the BMP-M group induced mature lamellar bone with well-organized trabeculae and minimal inflammatory infiltration by 16 weeks. However, the BMP-H group, despite high bone volume, exhibited residual necrotic fragments and mild inflammatory cell infiltration. Masson’s trichrome staining (Figure 3B) corroborated these findings: in the BMP-M group, disorganized blue-stained collagen was largely replaced by red-stained mineralized bone by 8 weeks, forming mature cortical structures by 16 weeks. Conversely, the BMP-H group retained more blue-stained collagen, indicating slower remodeling compared to the BMP-M group. These results suggest that 10 μg mL^-1^ rhBMP-2 may provide a more favorable balance between bone quantity and structural quality under the present experimental conditions.

### 3.4 Spatiotemporal expression of osteogenic markers

**FIGURE 4:**
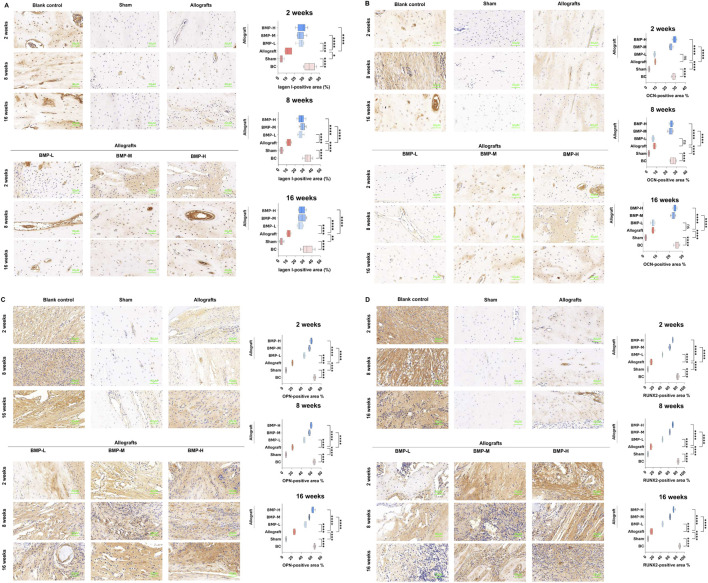
Immunohistochemical analysis of osteogenic markers during radial defect repair. Representative immunohistochemical staining and semiquantitative analysis of collagen I **(A)**, osteocalcin **(B)**, osteopontin **(C)**, and RUNX2 **(D)** in the blank control, sham, allograft, BMP-L, BMP-M, and BMP-H groups at 2, 8, and 16 weeks. Brown staining indicates positive expression of the corresponding marker. Semiquantitative analysis was performed as positive staining area (%) for all markers. Scale bars = 80 μm. Data are presented as mean ± SEM. ns, not significant; *P < 0.05; **P < 0.01; ***P < 0.001; ****P < 0.0001. BMP-L, BMP-M, and BMP-H correspond to 5, 10, and 20 μg mL^-1^ rhBMP-2, respectively.

IHC analysis quantified the expression of RUNX2, COL-I, OPN, and OCN (Figure 4).

Early Markers: At 2 weeks, RUNX2 expression peaked in the BMP-M and BMP-H groups, covering ∼80% of the graft area (*P* < 0.0001 vs. control) (Figure 4D).

Matrix/Mineralization Markers: COL-I and OPN expression expanded dose-dependently. The BMP-H group maintained significantly higher OPN levels through 16 weeks (Figures 4A,C).

Maturity Marker: OCN expression peaked at 8 weeks. Overall, the protein expression profiles reached a plateau at 10 μg mL^-1^. Increasing the dose to 20 μg mL^-1^ did not significantly further activate these osteogenic signaling pathways, providing molecular evidence for the saturation effect observed in Micro-CT.

### 3.5 Systemic inflammatory and repair cytokine dynamics

All rhBMP-2 groups showed suppressed acute inflammatory peaks (CRP and IL-6) compared to the unloaded allograft group, with the 10 and 20 μg mL^-1^ doses achieving a ∼50% reduction by day 7 (Tables 1, 2). Serum levels of CRP, IL-10, IL-6, BMP-2, TNF-α, IL-1β, and TGF-β1 are summarized in Tables 1–7, respectively. Compared with the unloaded allograft group, the rhBMP-2-treated groups showed lower acute inflammatory responses, particularly in CRP and IL-6 levels. The BMP-H group exhibited a secondary increase in pro-inflammatory TNF-α and IL-1β during the later inflammatory phase, indicating a risk of late-stage inflammatory rebound. Reparative and anti-inflammatory cytokines, including IL-10 and TGF-β1, showed time- and dose-dependent changes. While both 10 and 20 μg mL^-1^ doses upregulated anti-inflammatory factors (IL-10 and TGF-β_1_), the IL-10/IL-6 ratio-a marker of systemic homeostasis-remained most stable in the BMP-M group (Tables 5, 7). These findings suggest that 10 μg mL^-1^ rhBMP-2 may offer the most favorable balance between systemic inflammation suppression and regenerative signaling.

**TABLE 1:** The serum levels of CRP in different groups at different times.

Group	One day before first surgery	Next day after first surgery	One day before secondary surgery	Next day after secondary surgery	2 weeks	4 weeks	8 weeks
Sham	1.31 ± 0.02^***^	9.02 ± 0.21^***^	1.79 ± 0.03^***^	1.72 ± 0.03	1.81 ± 0.04^***^	1.55 ± 0.02^**^	1.45 ± 0.01
Allograft	0.93 ± 0.02^###***^	7.74 ± 0.09^###***^	3.22 ± 0.10^###***^	8.52 ± 0.02^###***^	4.91 ± 0.08^###***^	3.62 ± 0.04^###***^	1.33 ± 0.05^***^
Allograft + BMP2-A (5 μg/L)	1.10 ± 0.03***	5.84 ± 0.17^###***^	5.57 ± 0.08^###***^	6.09 ± 0.08^###***^	3.90 ± 0.05^###***^	2.85 ± 0.06^###***^	1.33 ± 0.02^***^
Allograft + BMP2-A (10 μg/L)	0.75 ± 0.01^###***^	6.07 ± 0.15^##^	5.92 ± 0.12^##^	5.12 ± 0.05^###***^	5.81 ± 0.09^###***^	3.07 ± 0.04^##***^	1.11 ± 0.02^#***^
Allograft + BMP2-A (20 μg/L)	1.72 ± 0.02^###***^	8.46 ± 0.12^###^	5.34 ± 0.03^###^	6.63 ± 0.03^###***^	4.04 ± 0.06^###***^	2.75 ± 0.04^###***^	2.28 ± 0.02^###***^
F	924.61	264.47	1487.23	8568.74	1452.94	1020.11	858.81
*P* value	8.54E-13	4.29E-10	7.97E-14	1.27E-17	8.96E-14	5.23E-13	1.23E-12

* indicates that in the same point, compared to the previous group, *P < 0.05, **P < 0.01, ***P < 0.001. # indicates that in the same group, compared to the previous time point, #P < 0.05, ##P < 0.01, ###P < 0.001.

**TABLE 2:** The serum levels of cytokines IL-10 in different groups at different times.

Group	One day before first surgery	Next day after first surgery	One day before secondary surgery	Next day after secondary surgery	2 weeks	4 weeks	8 weeks
Sham	34.11 ± 0.89	170.57 ± 2.89	42.76 ± 0.23	121.26 ± 0.46	15.79 ± 0.47	14.84 ± 0.12	29.59 ± 0.25
Allograft	21.68 ± 0.25	90.65 ± 2.12	93.01 ± 1.25	19.79 ± 3.40	113.37 ± 1.43	71.63 ± 0.13	34.18 ± 0.22
Allograft + BMP2-A (5 μg/L)	21.31 ± 0.26	83.81 ± 0.04	50.29 ± 1.13	106.79 ± 2.77	56.47 ± 0.66	89.17 ± 2.26	50.28 ± 0.63
Allograft + BMP2-A (10 μg/L)	35.37 ± 1.05	157.35 ± 2.32	36.94 ± 0.45	199.46 ± 6.58	100.23 ± 1.35	24.08 ± 0.78	38.69 ± 0.79
Allograft + BMP2-A (20 μg/L)	17.48 ± 0.41	151.94 ± 1.37	116.45 ± 4.33	195.60 ± 0.78	125.40 ± 0.47	64.10 ± 0.91	45.24 ± 0.78
F	456.78	1213.15	839.54	1547.95	6536.62	2321.84	593.13
P value	2.85E-11	2.20E-13	1.38E-12	6.53E-14	4.90E-17	8.63E-15	7.79E-12

**TABLE 3:** The serum levels of cytokines IL-6 in different groups at different times.

Group	One day before first surgery	Next day after first surgery	One day before secondary surgery	Next day after secondary surgery	2 weeks	4 weeks	8 weeks
Sham	50.59 ± 0.75	202.14 ± 6.22	36.99 ± 0.74	5.62 ± 0.56	27.37 ± 0.54	49.56 ± 0.91	17.31 ± 0.22
Allograft	36.74 ± 0.30	173.32 ± 2.07	145.52 ± 2.80	12.89 ± 3.10	124.84 ± 0.97	84.59 ± 0.28	42.23 ± 1.00
Allograft + BMP2-A (5 μg/L)	13.95 ± 0.20	178.92 ± 1.66	43.50 ± 0.51	189.86 ± 2.00	61.68 ± 0.51	101.65 ± 3.45	28.97 ± 0.93
Allograft + BMP2-A (10 μg/L)	49.07 ± 1.63	139.95 ± 2.78	76.87 ± 1.05	128.77 ± 1.92	36.10 ± 1.71	32.00 ± 0.44	36.20 ± 0.26
Allograft + BMP2-A (20 μg/L)	12.64 ± 0.40	187.40 ± 3.67	118.62 ± 1.57	95.04 ± 2.39	94.08 ± 1.70	58.06 ± 1.29	28.58 ± 0.15
F	1453.67	119.33	2724.69	3310.51	3392.72	793.82	657.28
P value	8.94E-14	2.15E-08	3.88E-15	1.47E-15	1.30E-15	1.83E-12	4.67E-12

**TABLE 4:** The serum levels of cytokines BMP2 in different groups at different times.

Group	One day before first surgery	Next day after first surgery	One day before secondary surgery	Next day after secondary surgery	2 weeks	4 weeks	8 weeks
Sham	110.37 ± 1.99	144.83 ± 1.19	140.91 ± 1.40	115.72 ± 0.68	135.07 ± 1.69	95.41 ± 1.77	69.44 ± 1.10
Allograft	56.68 ± 0.34	157.24 ± 1.24	96.88 ± 0.30	87.04 ± 0.89	98.83 ± 1.28	132.68 ± 2.39	133.93 ± 2.45
Allograft + BMP2-A (5 μg/L)	55.05 ± 0.74	251.90 ± 4.46	218.35 ± 1.74	140.33 ± 1.55	328.28 ± 4.02	287.70 ± 2.02	166.20 ± 6.35
Allograft + BMP2-A (10 μg/L)	109.60 ± 2.11	300.58 ± 4.82	245.05 ± 4.46	374.93 ± 1.40	795.32 ± 6.93	296.02 ± 2.17	195.34 ± 4.75
Allograft + BMP2-A (20 μg/L)	112.81 ± 2.19	289.70 ± 2.25	183.71 ± 1.37	311.26 ± 3.04	1063.58 ± 13.10	591.59 ± 6.18	134.36 ± 5.34
F	984.53	1581.11	1973.89	34,687.82	11,351.66	10,292.59	334.51
P value	6.24E-13	5.88E-14	1.94E-1	1.17E-20	3.10E-18	5.07E-18	1.34E-10

**TABLE 5:** The serum levels of cytokines TNF-α in different groups at different times.

Group	One day before first surgery	Next day after first surgery	One day before secondary surgery	Next day after secondary surgery	2 weeks	4 weeks	8 weeks
Sham	16.33 ± 0.35	131.06 ± 0.89	50.52 ± 0.72	45.24 ± 1.22	13.87 ± 0.43	18.63 ± 0.27	19.39 ± 1.01
Allograft	13.45 ± 0.75	206.36 ± 1.50	97.98 ± 2.57	203.99 ± 3.05	89.46 ± 2.07	90.35 ± 1.38	30.98 ± 0.35
Allograft + BMP2-A (5 μg/L)	43.22 ± 1.11	173.20 ± 3.28	106.19 ± 1.91	156.80 ± 1.10	104.58 ± 1.95	62.09 ± 1.26	39.17 ± 0.95
Allograft + BMP2-A (10 μg/L)	29.64 ± 0.67	100.89 ± 2.15	67.63 ± 0.37	76.38 ± 1.84	60.49 ± 2.13	97.11 ± 1.22	20.77 ± 0.46
Allograft + BMP2-A (20 μg/L)	33.54 ± 0.64	139.00 ± 4.16	148.90 ± 00.90	52.16 ± 0.36	92.44 ± 0.56	38.66 ± 0.69	31.51 ± 0.31
F	824.16	693.33	1847.47	4743.70	1516.62	3026.42	431.32
P value	1.51E-1	3.58E-12	2.70E-14	2.43E-16	7.23E-14	2.30E-15	3.80E-11

**TABLE 6:** The serum levels of cytokines IL-1β in different groups at different times.

Group	One day before first surgery	Next day after first surgery	One day before secondary surgery	Next day after secondary surgery	2 weeks	4 weeks	8 weeks
Sham	35.32 ± 1.13	155.54 ± 3.18	43.22 ± 0.25	32.26 ± 1.07	47.83 ± 0.75	23.15 ± 0.15	13.37 ± 0.16
Allograft	46.09 ± 0.17	193.19 ± 3.18	135.82 ± 0.88	149.02 ± 1.02	143.42 ± 1.41	30.03 ± 0.21	34.20 ± 0.66
Allograft + BMP2-A (5 μg/L)	38.11 ± 1.16	91.55 ± 1.34	63.79 ± 0.95	168.89 ± 2.67	62.58 ± 1.23	82.12 ± 0.42	19.03 ± 0.31
Allograft + BMP2-A (10 μg/L)	31.41 ± 0.36	91.74 ± 1.96	89.50 ± 0.55	78.15 ± 1.29	104.34 ± 11.38	90.51 ± 0.47	21.96 ± 0.81
Allograft + BMP2-A (20 μg/L)	41.69 ± 0.38	84.35 ± 0.99	73.94 ± 2.61	63.29 ± 1.94	124.07 ± 0.77	96.00 ± 0.12	50.49 ± 0.85
F	165.82	1986.90	2058.78	3452.98	3740.42	37,814.08	1700.31
P value	4.28E-09	1.88E-14	1.57E-14	1.19E-15	7.97E-16	7.58E-21	4.09E-1

**TABLE 7:** The serum levels of cytokines TGF-β in different groups at different times.

Group	One day before first surgery	Next day after first surgery	One day before secondary surgery	Next day after secondary surgery	2 weeks	4 weeks	8 weeks
Sham	415.97 ± 10.49	837.79 ± 7.92	335.97 ± 3.97	187.04 ± 2.75	327.04 ± 7.85	452.93 ± 8.22	226.72 ± 2.86
Allograft	133.71 ± 2.53	934.62 ± 15.68	659.66 ± 15.38	944.65 ± 12.89	539.15 ± 9.51	710.86 ± 3.16	210.34 ± 5.08
Allograft + BMP2-A (5 μg/L)	447.69 ± 0.59	875.59 ± 21.80	537.98 ± 18.12	578.82 ± 3.94	855.52 ± 15.02	432.15 ± 6.50	506.52 ± 11.69
Allograft + BMP2-A (10 μg/L)	482.38 ± 8.80	841.02 ± 14.17	764.32 ± 8.58	823.10 ± 2.16	327.75 ± 7.81	689.18 ± 5.75	428.21 ± 3.41
Allograft + BMP2-A (20 μg/L)	335.87 ± 3.23	795.40 ± 27.90	494.62 ± 10.62	901.36 ± 5.96	316.23 ± 7.58	632.74 ± 7.46	193.24 ± 3.01
F	1421.48	22.95	521.15	6436.93	1645.01	1254.58	1628.59
P value	9.99E-14	5.04E-05	1.48E-11	5.29E-1	4.82E-14	1.86E-13	5.07E-14

## 4 Discussion

In this study, we utilised a 1.5 cm rabbit radial defect model to demonstrate that rhBMP-2 exhibits a distinct ‘threshold-saturation’ effect when integrated within the MIMT framework. Our results suggest that, within the tested dosing range, 10 μg/mL may represent the most favorable dose, as evidenced by a plateau in bone volume fraction (BV/TV), radiographic healing scores and the expression of key osteogenic markers (RUNX2, COL-I, OPN and OCN). Although the 20 μg/mL dose produced a modest (∼10%) increase in bone volume, it did not result in any substantial improvements in bone maturity or systemic inflammatory stability. These findings support a non-linear dose-response relationship and suggest that further dose escalation may not necessarily improve regenerative quality (Kamal et al., 2019; Decambron et al., 2017).

A central premise of this investigation was the potential interaction between the induced membrane and rhBMP-2. Distinct from conventional bone tunnel or ectopic models, the MIMT establishes a localized “biological bioreactor” characterized by a robust angiogenic-osteogenic niche, abundant in vascularization and osteoprogenitor recruitment factors such as VEGF and IL-6 (Aho et al., 2013). Our immunohistochemical (IHC) data revealed a significant upregulation of RUNX2 expression within the membrane chamber compared to the pure allograft group, suggesting that the membrane microenvironment may enhance BMP-2-mediated signaling. Furthermore, while sophisticated controlled-release scaffolds are frequently explored in experimental settings (Bosemark et al., 2015). Our approach—utilizing freeze-dried allografts as delivery vehicles—demonstrates a cost-effective and clinically viable strategy that facilitates direct translational application in complex reconstructive surgeries.

An important observation in this study was the spatiotemporal monitoring of systemic inflammatory dynamics. Although rhBMP-2 is indispensable for osteoinduction, the application of supra-physiological dosages is well-documented to elicit exaggerated inflammatory responses and localized soft tissue edema. Our findings indicate that 10 μg mL^-1^ rhBMP-2 effectively attenuated the acute inflammatory peaks of CRP and IL-6 by approximately 50%, while maintaining a favorable IL-10/IL-6 ratio—a critical marker of systemic immune homeostasis. In contrast, the 20 μg mL^-1^ group exhibited a secondary pro-inflammatory resurgence (characterized by elevated TNF-α and IL-1β) at day 14. Histological analysis corroborated this systemic “rebound,” revealing residual necrotic bone fragments and persistent inflammatory infiltration. This mirrors the findings of Zara et al., who cautioned that excessive BMP-2 (≥40 μg mL^-1^) may precipitate maladaptive “cystic” bone formation and compromised structural remodeling (Zara et al., 2011). Collectively, these data underscore that biological quality and structural maturity, rather than absolute bone volume, must serve as the primary benchmarks for evaluating successful skeletal reconstruction.

Furthermore, to the best of our knowledge, this study provides a spatiotemporal characterization of osteogenic protein dynamics (RUNX2, COL-I, OPN, and OCN) under a rhBMP-2 concentration gradient within the MIMT framework. The sequential transition from early-phase RUNX2 peaking to late-stage OCN synthesis aligns with the established hierarchy of osteoblast lineage commitment (Tang et al., 2020; Shu et al., 2021), suggesting that 10 μg mL^-1^ may be sufficient to support key stages of the osteogenic cascade of both intramembranous and endochondral ossification (He et al., 2013). The robust expression profiles observed at this dosage-characterized by RUNX2 levels exceeding 80% and OCN levels surpassing 50%-indicate that the regenerative response of the defect site may have approached a plateau under these conditions. Accordingly, further dose escalation beyond this level may offer limited additional biological benefit; beyond this threshold, higher concentrations do not further enhance lineage commitment but instead may become counterproductive, as evidenced by the concurrent systemic inflammatory perturbations and compromised bone maturity.

In this study, the secondary inflammatory rebound observed in the BMP-H group at day 14 (T_8_) may represent a cautionary signal in BMP-2 application. While conventional dose-response models primarily focus on the plateauing of osteogenic efficacy (Wei et al., 2012), our data suggest the presence of a potential pro-inflammatory threshold where the anabolic benefits of rhBMP-2 are effectively neutralized by iatrogenic immunomodulatory dysregulation. The recrudescence of pro-inflammatory cytokines (TNF-α and IL-1β), coupled with late-stage histological evidence of necrotic residues and persistent inflammatory infiltration at 16 weeks, suggests that supra-physiological concentrations may precipitate a maladaptive remodeling cycle (James et al., 2016). This phenomenon aligns with previous reports indicating that excessive BMP-2 can trigger uncontrolled osteoclast activation and soft tissue edema, ultimately compromising the quality of the regenerated bone (Shields et al., 2006). Mechanistically, the inflammatory peak observed at day 14 likely reflects a delayed immune mobilization in response to the rapid, rhBMP-2-induced recruitment of osteoclast precursors (Muto et al., 2011) or the metabolic exhaustion of over-stimulated mesenchymal progenitors. In contrast to the BMP-M group, which maintained a stable 1L-10/IL-6 ratio—indicative of resolved inflammation and systemic homeostasis, the BMP-H group appeared to show a less coordinated transition from the inflammatory phase to the remodeling phase. This apparent mismatch between inflammatory resolution and tissue remodeling was accompanied histologically by residual necrotic tissue, which we hypothesize results from impaired vascular-bone coupling. Specifically, supra-physiological concentrations of rhBMP-2 may drive osteogenesis so aggressively that the localized angiogenic response cannot satisfy the metabolic demands of the rapidly expanding bone volume, leading to intramedullary hypoxia and the formation of ‘cystic’ or ischemic trabecular cores (Aghaloo et al., 2007).

These findings suggest a need to refine current clinical strategies that rely on escalating growth factor dosages to address extensive segmental defects (James et al., 2016; Carragee et al., 2011). Our data indicate that the relationship between dosage and bone healing is characterized by a complex, non-linear interplay rather than a simple dose-response model, highlighting that successful regeneration depends as much on maintaining immunomodulatory homeostasis as on the magnitude of the osteogenic stimulus (Yang and Liu, 2021; Tsukasaki and Takayanagi, 2019). While acknowledging the clinical necessity of potent osteoinductive agents for massive bone loss, our results suggest that an immuno-favorable environment, rather than supra-physiological induction, may be an important prerequisite for high-quality bone regeneration (Ward et al., 2025). As a structural bioreactor characterised by its rich vascularisation and endogenous growth factor secretion, the induced membrane may inadvertently amplify the systemic inflammatory risks of high-dose rhBMP-2. This may render the system more sensitive to dosage-dependent complications than standard grafting procedures (Gindraux et al., 2017; Alford et al., 2021).

From a clinical perspective, the safety profile of rhBMP-2 remains a major concern, with reported complication rates of 10%–50% in spinal fusions involving high-dose applications (Carragee et al., 2011). By utilizing a moderate-load radial defect model, we provided a more realistic Inter-species dose-conversion benchmark than previous calvarial models. The absence of ectopic ossification and the relative maintenance of systemic homeostasis in the BMP-M group suggest a comparatively favorable safety profile for treating long bone defects.

There are several limitations to this study. First, although the 16-week follow-up was sufficient to assess bridging, it did not evaluate long-term biomechanical strength (e.g., torsional or bending properties) or the ultimate fate of the induced membrane. Second, we did not measure *in vivo* release kinetics; therefore, the precise local concentration of BMP-2 over time remains uncertain. In addition, rhBMP-2 amounts were reported as nominal/theoretical amounts based on the standardized defect volume, because adsorption efficiency and retained loading after soaking and vacuum drying were not directly quantified. Future studies should investigate ultra-low dose gradients (<5 μg mL^-1^) and low-peak, sustained-release delivery systems. Furthermore, integrating spatial transcriptomics may help to further dissect the osteo-immune-vascular crosstalk within the induced membrane microenvironment at higher resolution. Finally, multicenter clinical studies will be required to determine whether the apparent 10 μg mL^-1^ plateau observed in this rabbit model can be translated to human critical-sized bone defects.

Future studies should employ single-cell RNA sequencing (scRNA-seq) to further elucidate whether this inflammatory rebound is driven by a specific subpopulation of M1-polarized macrophages within the induced membrane environment.

## 5 Conclusion

This study demonstrates that rhBMP-2 exhibits a “threshold-saturation” effect within the MIMT framework. While both 10 and 20 μg mL^-1^ doses effectively promote bone bridging and suppress acute systemic inflammation, within the tested conditions, the 10 μg mL^-1^ concentration appeared to provide the most favorable balance between osteogenesis and systemic immune homeostasis. This concentration was associated with reduced inflammatory fluctuation and fewer histological abnormalities than the higher-dose group. These findings provide experimental support for further optimization of BMP-2 dosing in MIMT-based bone reconstruction.

## Data Availability

The raw data supporting the conclusions of this article will be made available by the authors, without undue reservation.
